# Prognosis of STEMI Patients with Multi-Vessel Disease Undergoing Culprit-Only PCI without Significant Residual Ischemia on Non-Invasive Stress Testing

**DOI:** 10.1371/journal.pone.0138474

**Published:** 2015-09-25

**Authors:** Adaya Weissler-Snir, Chen Gurevitz, Abid Assali, Hana Vaknin-Assa, Tamir Bental, Adi Lador, Hagai Yavin, Leor Perl, Ran Kornowski, Eli Lev

**Affiliations:** 1 Cardiology Department, Rabin Medical Center, Petah-Tikva, Israel; 2 The Sackler Faculty of Medicine, Tel-Aviv University, Tel Aviv, Israel; Niigata University Graduate School of Medical and Dental Sciences, JAPAN

## Abstract

**Aims:**

In about 50–80% of ST-segment elevation myocardial infarction (STEMI) patients there is significant atherosclerotic disease in other coronary arteries in addition to the culprit vessel. There is substantial controversy as to the optimal revascularization approach in these patients. We sought to compare the outcomes of STEMI patients with multi-vessel disease (MVD) treated with culprit-only primary percutaneous coronary intervention (PPCI) without significant ischemia on subsequent non-invasive testing, to those of STEMI patients with single-vessel disease (SVD).

**Methods and Results:**

Between 2001–2010, 1,540 consecutive patients treated with primary PCI for STEMI were prospectively observed and entered into a comprehensive clinical database. The primary end point was a composite of major adverse cardiac events (MACE), consisting of mortality, re-infarction and revascularization within 1 and 3 years following PPCI (excluding events occurring during the first 30 days). Patients with cardiogenic shock were excluded. The study included 720 patients with SVD and 185 patients with MVD who underwent culprit-only PPCI and had no residual ischemia on subsequent non-invasive stress testing. Patients with MVD were older, more likely to have hypertension or previous MI and less likely to be smokers and present with anterior MI than patients with SVD. One and 3-year MACE rates were similar between the groups. On cox proportional-hazards regression MVD without residual ischemia was not independently associated with MACE and its components.

**Conclusions:**

STEMI patients with MVD treated with culprit only-PCI without significant residual ischemia on non-invasive stress testing appear to have similar prognosis to STEMI patients with SVD.

## Introduction

The main goal of primary percutaneous coronary intervention (PCI) in the setting of ST elevation myocardial infraction (STEMI) is to re-perfuse the myocardium by opening the culprit (infarct-related) coronary artery. However, in as many as 50–80% of patients with STEMI there is significant atherosclerotic disease in other coronary arteries in addition to the culprit vessel; a state which is associated with adverse outcomes [1–7]. Many studies have examined the value of an early treatment strategy performed upon significant narrowing in the non-culprit vessels during primary PCI [3–14]. Most studies have shown that such intervention does not provide clinical benefit, and at times may even be harmful [3–5,8–14]. By contrast, two recent randomized studies did show benefit for a strategy of non-culprit PCI during the index procedure [14,15]. Despite these two studies, postponing and staging possible treatment of the non-culprit vessels is still the preferable approach by current guidelines on myocardial revascularization and STEMI [16–18]. However, the clinical strategy for staging or postponing PCI in these patients is debatable. Several expert opinions recommend routine elective PCI of significant stenoses in non-culprit vessels several days or weeks after the primary PCI, regardless of clinical features or further studies. Other experts promote performance of non-invasive provocative testing to assess ischemia in the non-culprit territories, such as an exercise nuclear scan or a stress echocardiogram, in order to determine the necessity of performing PCI of the non-culprit vessels [4,13,19]. The latter approach is based on the assumption that during acute MI, due to the release of cytokines and vasoactive agents to the circulation, a temporary vasoconstriction of non-culprit vessels may occur, which may overstress severity and resolve spontaneously after the recovery of the acute phase [5,20].

A central question that may guide decision-making in this controversial issue is what the prognosis is of patients with STEMI with multi-vessel disease undergoing culprit vessel PCI without significant ischemia on subsequent non-invasive testing (and thus they did not undergo further PCI).

We hypothesized that the prognosis of these patients is similar to that of patients with single vessel disease undergoing successful primary PCI. Hence, the aim of this study was to compare the prognosis of these two groups of patients.

## Materials and Methods

Between January 2001 and December 2010, 1,540 consecutive patients with STEMI undergoing primary PCI at the Rabin Medical Center, Israel, were prospectively observed and entered into a comprehensive clinical database. Acute STEMI was defined as the presence of typical chest pain and accompanying symptoms for a duration of at least 30 minutes but < 12 hours in the presence of ST-segment elevation ≥1 mm in at least 2 contiguous leads, or new or undetermined duration of left bundle branch block in association with elevated cardiac enzymes (Creatine kinase, Troponin I or T). The registry included demographic, clinical, angiographic, procedural and echocardiographic data. This registry was approved by the ethics committee of the Rabin Medical Centre, in compliance with the Declaration of Helsinki.

Patient records/information was anonymized and de-identified prior to analysis.

All patients were treated with aspirin 300 mg before the PCI and clopidogrel 600 mg. Unfractionated heparin (70 U/Kg loading) was given before PCI and adjusted to achieve an activated clotting time of 200 to 275 seconds during the intervention. Glycoprotein IIb/IIIa receptor inhibition by eptifibatide was used at the discretion of the operator only after crossing the culprit lesion with a guidewire. Coronary angiography was performed through the femoral or radial rout according to the operator’s discretion. All patients had PCI performed only to the culprit vessel according to the institutional policy at the time of data collection (2001–2010). Selection of stent type, pre-dilatation with undersized balloons, and post-dilatation with larger balloons also were left to the operator’s discretion. Procedural success was defined as an angiographic residual stenosis of 20% or less by visual estimation or quantitative coronary angiography with optimized angiographic flow (Thrombolysis In Myocardial Infarction (TIMI) flow 3). All patients were prescribed lifelong aspirin and clopidogrel (75 mg daily) for 12 months. Baseline clinical characteristics, angiographic details, quantitative coronary angiography, TIMI flow and clinical outcomes were collected.

Patients were stratified according to the presence or absence of multi-vessel disease, which was defined as ≥ 70% stenosis of ≥ 2 epicardial coronary arteries or their major branches. Patients with multi-vessel disease were further classified according to whether or not they had undergone staged PCI to the non-culprit vessels based on the results of non-invasive stress testing for residual ischemia performed 4–6 weeks after the index PCI. The test was considered diagnostic when heart rate reached at least 85% of target heart rate.

Exclusion criteria for the current analysis were:

1. Cardiogenic shock at presentation
2. Previous coronary artery bypass grafting (CABG)
3. Death, revascularization or re-infarction within 1 month of the index event
4. Multivessel disease without non-invasive testing for residual ischemia or with findings of significant ischemia following the index procedure

The study primary endpoint was the 1-year and 3-year cumulative rates of major adverse cardiovascular events (MACE) defined as a composite of all-cause mortality, revascularization (i.e. coronary artery bypass grafting and/or catheter-based target vessel revascularization) and re-infarction (excluding events occurring during the first 30 days after the index PCI). Secondary endpoints included the rates of individual components of MACE at 1 and 3-years.

All events were further adjudicated by a research coordinator and reviewed by an experienced cardiologist from our research team. For each patient, a standardized questionnaire was completed either by telephone or in the outpatient clinic at 1, 6, 12, 24 and 36-month follow-ups. Mortality was confirmed by the records of the Interior Ministry of Israel. Repeat revascularization procedures and episodes of reinfarction were confirmed using the hospital as well as affiliated hospitals databases. These databases were searched for all patients in the study to gather information regarding repeat events. The diagnosis of reinfarction during follow-up was based on recurrent chest pain, suggestive of acute MI, accompanied by re-elevation of the cardiac enzyme with at least one value above the 99th percentile upper reference limit at least 48 hours after PCI and/or new ST elevation, new left bundle branch block or development of pathological Q waves in the electrocardiogram. Target vessel revascularization was defined as any revascularization that involved the target vessel. Stent thrombosis was defined according to the Academic Research Consortium definitions as “definite” in the context of acute coronary syndrome and/or reinfarction in the culprit coronary territory with angiographically proven thrombosis (thrombus or occlusion) of the previously implanted stent.

## Statistical Analysis

Data are presented as mean ± standard deviation for normally distributed variables and as median (interquartile range (IQR)) for non-normally distributed variables. Continuous variables were compared using Student’s *t* testing or Mann Whitney testing, as appropriate. Categorical variables were compared using chi-square statistics or Fischer’s exact testing, as appropriate.

Time-to-event curves using the Kaplan-Meier method were calculated and compared using with the Log-Rank testing. Cox proportional hazards regression model using the enter method to control for confounders that are expected to be related to long-term outcomes (age, sex, glomerular filtration rate, diabetes mellitus, left anterior descending artery disease, pre-TIMI flow grade<2 and left ventricle ejection fraction) was performed.

All tests were two-tailed, and a P-value <0.05 was considered significant. Analyses were performed using SPSS 21.0 statistical software package (IBM SPSS Inc).

## Results

**Fig 1:**
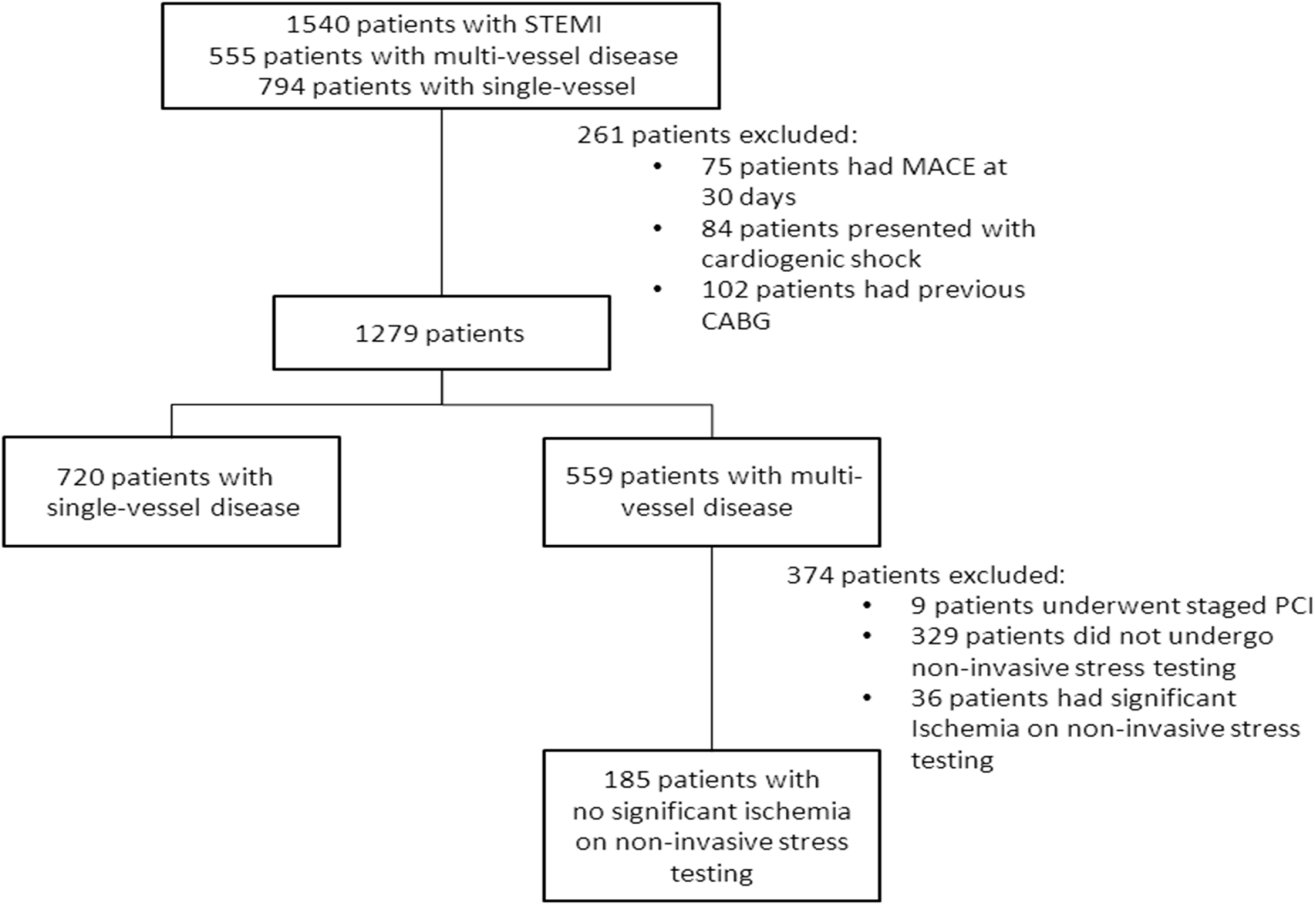
Study cohort. Baseline and angiographic characteristics of the patients with multi-vessel disease vs. patients with single-vessel disease are summarized in Table 1.

A total of 905 patients were included in the present analysis, of whom 720 had single-vessel disease and 185 had multi-vessel disease (73 with 3-vessel disease) without evidence of significant residual ischemia on subsequent non-invasive stress testing (Fig 1).

**Table 1:** Baseline and angiographic characteristics of patients with multivessel disease vs. patients with single-vessel disease.

	Single-vessel disease (n = 720)	Multivessel disease (n = 185)	P value
Mean age (± SD), years	57 ± 12	62 ± 10	<0.001
Male, %	80.7	85.4	0.14
GFR, ml/min	92±27	86±26	0.63
GFR <60ml/min, %	9	12.8	0.12
Diabetes Mellitus, %	21.9	27.7	0.11
Hypertension, %	40.3	53.8	0.001
Dyslipidemia, %	46.5	52.7	0.13
Smoking, %	62.4	51.1	0.007
Current smoker, %	51.2	38.3	
Past smoker, %	11.2	12.8	
History of MI, %	6.5	13.1	0.015
History of angioplasty, %	9.8	17.1	<0.001
Previous CVA, %	3.2	6.1	0.06
Peripheral vascular disease, %	3.5	3.4	0.94
Killip class≥2, %	10.3	14	0.15
Ejection fraction≤40%, %	39.2	38.3	0.86
Infarct location, %			0.005
Anterior	53.3	40	
Inferior	41.7	54.1	
Lateral	5	5.9	
LAD culprit	55.7	40	0.001
Pre-TIMI Flow 0	56.5	60	0.4
Post-TIMI Flow 3	95.6	96.2	0.66
Thrombus aspiration, %	17.2	14.5	0.19
Glycoprotein IIb/IIIa inhibitors, %	74.8	77.3	0.31

CVA = Cerebrovascular accident, GFR = Glomerular filtration rate; MI = Myocardial infarction, LAD = Left anterior descending artery; TIMI = Thrombolysis In Myocardial Infarction

Patients with multi-vessel disease were older than patients with single vessel disease (mean difference of 4.8 years, p<0.001). They were more likely to have hypertension and less likely to be current or ex-smokers than patients with single-vessel disease. Previous MI was more prevalent amongst patients with multi-vessel disease than in patients with single-vessel disease (13.1% vs. 6.5%, p = 0.015). A greater proportion of patients with single-vessel disease presented with anterior MI as compared with patients with multi-vessel disease (53.3% vs. 40.0%, p = 0.005). Neither the Killip class nor the left ventricular systolic function differed significantly between the 2 groups.

**Fig 2:**
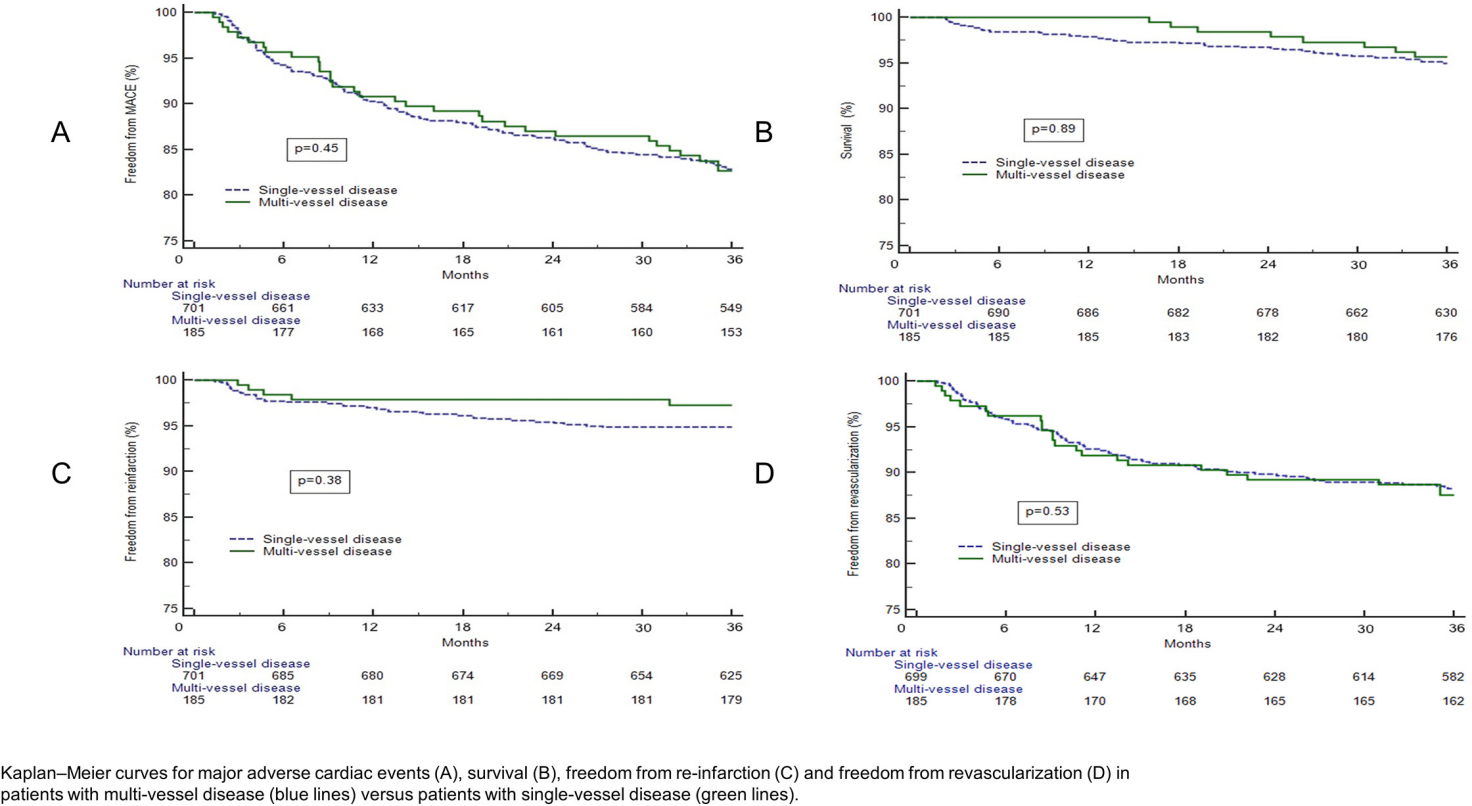
Clinical Outcomes of Patients with Multi-Vessel Disease vs. Patients with Single-Vessel Disease. Kaplan–Meier curves for major adverse cardiac events (A), survival (B), freedom from re-infarction (C) and freedom from revascularization (D) in patients with multi-vessel disease (blue lines) versus patients with single-vessel disease (green lines).

Mean follow up was 83.6 ± 32 months. Clinical outcomes are presented in Table 2, Fig 2.

**Table 2:** Outcomes of patients with single-vessel disease vs. patients with multi-vessel disease (excluding first 30 days post-PPCI).

1-year outcome (%)	Single-vessel disease	Multi-vessel disease	Log-Rank, P value
Mortality	2.5	0	0.03
Re-infarction	3.3	2.8	0.4
Revascularization	8.1	7.6	0.83
Major adverse cardiac events	10.9	8.6	0.39
3-year outcome (%)	Single-vessel disease	Multivessel disease	Log-Rank, P value
Mortality	5	5.4	0.89
Re-infarction	5.3	4.8	0.38
Revascularization	11.7	13.5	0.53
Major adverse cardiac events	17.1	20	0.45

MACE rates calculated by time to first event up to 1 and 3-years were similar between the 2 groups (Table 2, Fig 2A). There were no mortality events in the multi-vessel disease group during the first year after the index procedure (excluding the first 30 days) compared with a 2.5% mortality rate in the single-vessel disease group (p = 0.03). At 3-years after PPCI, mortality rate of patients with multi-vessel disease was similar to that of patients with single-vessel disease. Re-infarction and revascularization rates at 1 and 3-years did not differ between patients with single-vessel disease and those with multi-vessel disease (Fig 2B–2D).

On cox proportional hazards regression model, multi-vessel disease without residual ischemia was not independently associated with MACE and its components at 1 and 3-years, whereas diabetes was independently associated with both MACE at 1 and 3-years and female sex for MACE at 1-year (Tables 3 and 4).

**Table 3:** Multiple Cox regression models for the association between multi-vessel disease and 1 and 3-year outcomes (excluding first 30 days post-PPCI.

1-year Outcome	Unadjusted HR [95%] CI, P value	Adjusted* HR [95%] CI, P value
Mortality	N/A**	N/A**
Re-infarction	1.5 [0.52–4.38], P = 0.44	1.83 [0.62–5.4], P = 0.27
Revascularization	0.94 [0.52–1.68], P = 0.83	1.15 [0.63–2.1], P = 0.65
MACE	1.5 [0.88–2.65], P = 0.12	1.27 [0.71–2.23], P = 0.42
3-year Outcome	Unadjusted HR [95%] CI, P value	Adjusted* HR [95%] CI, P value
Mortality	1.03 [0.51–2.08], P = 0.92	0.74 [0.33–1.65], P = 0.46
Re-infarction	0.68 [0.31–1.54], P = 0.36	0.80 [0.34–1.86], P = 0.61
Revascularization	1.15 [0.72–1.76], P = 0.53	1.37 [0.86–2.19], P = 0.18
MACE	1.10 [0.78–1.63], P = 0.51	1.10 [0.8–1.75], P = 0.39

* Adjusted for age, sex, glomerular filtration rate, diabetes mellitus, left anterior descending artery disease, pre-TIMI flow grade<2 and left ventricle ejection fraction

** No mortality events in the multi-vessel group

CI = Confidence interval; HR = Hazard ratio; MACE = Major adverse cardiac events

**Table 4:** Multiple Cox regression for MACE at 1 and 3 years.

	Hazard Ratio	95% Confidence interval	P value	Hazard Ratio	95% Confidence interval	P value
Age	0.99	0.97–1.01	0.42	1.00	0.99–1.02	0.53
GFR	0.99	0.99–1.000	0.49	1.01	0.99–1.01	0.90
MVD	0.91	0.52–1.58	0.73	1.17	0.79–1.73	0.43
LAD culprit	1.07	0.52–1.58	0.79	1.08	0.75–1.57	0.66
Pre PCI TIMI grade flow<2	1.06	0.69–1.65	0.77	1.27	0.89–1.79	0.18
Diabetes	1.88	1.19–2.94	0.006	1.79	1.27–2.54	0.0007
Female sex	1.69	1.02–2.76	0.04	1.46	0.98–2.15	0.08
Ejection fraction during index hospitalization	0.99	0.97–1.02	0.49	0.99	0.97–1.01	0.46

GFR = Glomerular filtration rate; LAD = Left anterior descending artery; MACE = Major adverse cardiac events; MVD = Multi-vessel disease; PCI = Percutaneous coronary intervention; TIMI = Thrombolysis in Myocardial Infarction

Patients with adverse outcomes occurring during the first 30 days following the procedure were not included in the final analysis (as patients underwent non-invasive testing usually within 4 weeks of the index procedure). A sub-analysis of patients who experienced an adverse outcome during the first 30 days after the index event revealed that the MACE rates at 30 days did not differ significantly between patients with single-vessel disease and patients with multi-vessel disease treated with culprit-PCI only (4.6% vs. 6.4%, respectively, Log-Rank p = 0.13). Mortality rates at 30-days were higher in the multi-vessel group, as compared with the single vessel group (3.9% vs. 1.7%,) but after adjustment for confounders multi-vessel disease per se did not remain independently associated with mortality at 30-days.

## Discussion

Multi-vessel coronary artery disease is prevalent amongst patients undergoing primary PCI for STEMI [1,2]. While current guidelines recommend that in stable patients primary PCI should be limited to the culprit vessel, there has been an ongoing debate regarding the necessity and timing of revascularization of the non-culprit vessels after the acute event has resolved. Some authors advocate PCI of the non-infarct related vessels with the aim to achieve complete revascularization whenever possible. In contrast, others support ischemia-guided therapy in order to detect functionally significant lesions and to avoid unnecessary interventions. Several meta-analyses have shown that staged-PCI is associated with better outcomes compared with multi-vessel primary PCI and culprit-only PCI [21–23]. However none of these meta-analyses scrutinized the additive value of assessment of ischemia prior to staged-PCI.

We have found that patients with STEMI and multi-vessel coronary disease treated with primary PCI of the culprit vessel-only, who had not demonstrated significant residual ischemia on subsequent non-invasive stress testing, had similar outcomes at 1 and 3-years after the index event to those patients with single vessel disease. Interestingly, 1-year mortality rates were lower in the multi-vessel disease group than in the single-vessel disease group. A possible explanation is the exclusion of patients who experienced an adverse event within the first 30 days following the index event from the analysis.

We have also shown that patients with multi-vessel disease treated with culprit-only PCI had similar 30-day outcomes to patients with single vessel disease. These data may imply that it is safe to postpone stress testing following STEMI until a few weeks have elapsed.

Notably in the vast majority of patients with multi-vessel disease who had undergone non-invasive stress testing in our cohort (83.7%) the non-culprit arteries were not associated with significant residual ischemia on noninvasive stress testing performed 4–6 weeks after the index event. Indeed, Hanratty et al have previously shown that a significant exaggeration of non-culprit stenosis severity occurred at the acute MI angiography in 21% of patients [20]. Moreover, Dambrinkt et al have found that hemodynamic significance of non-culprit lesions, as detected by fractional flow reserve during primary PCI, was overestimated in 40% of the lesions [5]. Several hypotheses on the causes of the exaggeration in non-culprit lesions stenosis have been suggested including increased circulating catecholamine levels, enhanced bioactivity of important coronary vasoconstrictors, such as serotonin, endothelin, angiotensin, and thromboxane and reduced vasodilatory effects of nitric oxide, adenosine and prostacyclin [24–28].

It is noteworthy, that in contrast to the aforementioned studies, the recent PRAMI study, has found that preventive PCI at time of the index procedure (in culprit and non-culprit coronary arteries with significant stenosis) was superior to PCI limited to the culprit artery in reduction of MACE [14]. However, the study did not address the question of immediate preventive PCI during the index procedure versus staged PCI within days-weeks of the MI. Additionally, its relatively small sample size (n = 465) and premature cessation might cause the end points to appear more significant than they might be with a longer follow-up. Likewise, the CvLPRIT trial, has found that complete revascularization at the time of the PPCI or during the index hospitalization yielded better outcomes at 12 months than culprit-only PCI [15]. However, similar to the PRAMI trial this was a relatively small sized study (n = 278) that did not differentiate between an early staged vs. multivessel PCI strategies during the course of STEMI. Two meta-analyses of randomized controlled trials, conducted following the PRAMI and CvLPRIT trials by Elgendy et al and Dahal et al, suggested that multi-vessel PCI at the time of PPCI for STEMI resulted in better outcomes than culprit-vessel only PCI [29,30]. However, both included limited number of studies (i.e. 4 and 6) with a relatively small number of patients in each study except for the PARMI trial, which, thus, had the greatest weight of the sample size. Additionally, the design and follow-up time in the studies were variable. Furthermore, the meta-analysis by Elgendy et al. was underpowered for hard outcomes such as mortality and non-fatal MI [29]. Notably, although Dahal et al. found reduced MACE with multi-vessel PCI compared with culprit-only PCI, there was no significant difference in MACE between staged-PCI and multi-vessel PCI during the index procedure [30].

## Limitations

The current study has several limitations. First, it was a retrospective and nonrandomized single center study that was subjected to our local mode of practice. Second, only 40% of patients with multi-vessel disease underwent non-invasive stress testing. Third, we excluded patients who had an adverse event during the first 30 days following the index procedure, as patients underwent non-invasive testing usually within 4 weeks following the index procedure. Hence, the outcome analysis did not include events occurring up to 30 days after the index procedure. Information about the cause of death was not available; therefore we could only assess the rate of all cause mortality. Pervious studies have shown that approximately 30% of patients have residual stress defects following PCI and are known to be at higher risk of adverse events. However, since performing non-invasive stress testing following PPCI in patients with SVD is not common at our medical center we cannot rule out residual ischemia in patients with SVD.

## Conclusions

We have shown that patients with STEMI and multi-vessel disease treated with culprit-only PCI without significant residual myocardial ischemia on non-invasive stress testing have similar short- and long-term prognosis to STEMI patients with single vessel disease. These findings support a strategy of employing non-invasive stress testing for the assessment of ischemia in patients with STEMI and multi-vessel disease following the culprit artery PCI. Such strategy can guide the management and prevent unnecessary interventions in this STEMI patient population.

## Supporting Information

S1 File Baseline characteristics and outcomes of the final cohort. (XLS)

S1 Table Baseline characteristics of patients who underwent stress testing compared to patients who did not undergo stress testing. (DOCX)

## Abbreviations

MACE: Major adverse cardiac events
STEMI: ST-segment elevation myocardial infarction
PCI: Percutaneous coronary angioplasty
TIMI: Thrombolysis In Myocardial Infarction
