# Supplementary material for: Prognosis of STEMI Patients with Multi-Vessel Disease Undergoing Culprit-Only PCI without Significant Residual Ischemia on Non-Invasive Stress Testing
Source: PLoS One. 2015 Sep 25;10(9):e0138474. doi: 10.1371/journal.pone.0138474 (PMC4583296; doi:10.1371/journal.pone.0138474)
Supplement: S1 Table — (DOCX) [file pone.0138474.s002.docx]

S1 Table. Baseline characteristics of patients who underwent stress testing compared to patients who did not undergo stress testing

| Variable | Stress test (n=219) | No stress test (n=329) | P value |
| --- | --- | --- | --- |
| Mean age (± SD), years | 61.8 ± 10.4 | 63.4 ± 11.1 | 0.1 |
| Male, % | 86.7 | 83.7 | 0.39 |
| Hyperlipidemia, % | 50.7 | 52.1 | 0.47 |
| Previous CVA, % | 5.5 | 4.2 | 0.57 |
| Hypertension, % | 54.3 | 55.1 | 0.7 |
| Diabetes, % | 28.8 | 32.8 | 0.42 |
| Previous MI, % | 9.6 | 11.4 | 0.56 |
| Previous angioplasty, % | 14.6 | 16.9 | 0.55 |
| Smoking, % | 51.1 | 54.2 | 0.42 |
| Current smoker, % | 37.4 | 37.3 |  |
| Past smoker, % | 13.7 | 16.9 |  |
| GFR <60ml/min, % | 13.2 | 11.4 | 0.8 |
| GFR, ml/min | 85.6±26.8 | 87.7±27.2 | 0.38 |
| Peripheral vascular disease, % | 3.2 | 3.9 | 0.65 |
| Three-vessel disease, % | 42.0 | 41.2 | 0.93 |
| Infarct location |  |  | 0.77 |
| Anterior | 40.6 | 40.9 |  |
| Inferior | 53.8 | 52.1 |  |
| Lateral | 5.5 | 6.9 |  |
| Ejection fraction<40%, % | 40.6 | 37.3 | 0.08 |
| Ejection fraction, % | 42±10 | 43±10 | 0.63 |
| CVA = Cerebrovascular accident, GFR = Glomerular filtration rate; MI = Myocardial infarction | | | |
